# Factors associated with knowledge and awareness of stroke among the Lebanese population: A cross-sectional study

**DOI:** 10.12688/f1000research.108734.1

**Published:** 2022-04-14

**Authors:** Diana Malaeb, Nada Dia, Chadia Haddad, Souheil Hallit, Hala Sacre, Muna Barakat, Sara Mansour, Pascale Salameh, Hassan Hosseini

**Affiliations:** 1 School of Pharmacy, Lebanese International University, Beirut, Lebanon; 2 Life Sciences and Health Department, Paris-Est University, Paris, France; 3 College of Pharmacy, Gulf Medical University, Ajman, United Arab Emirates; 4 Research department, Psychiatric Hospital of the Cross, JalEddib, Lebanon; 5 INSERM, Univ. Limoges, CH Esquirol, IRD, U1094 tropical Neuroepidemiology, Institute of Epidemiology and Tropical Neurology, GEIST, Limoges, France; 6 School of Medicine and Medical Sciences, Holy Spirit University of Kaslik, Jounieh, P.O Box 446, Lebanon; 7 Epidémiologie Clinique et Toxicologie, INSPECT-LB: Institut National de Santé Publique, Beirut, Lebanon; 8 Department of Clinical Pharmacy and Therapeutics, Faculty of Pharmacy, Applied Science Private University, Amman, Jordan; 9 Faculty of Pharmacy, Lebanese University, Hadat, Lebanon; 10 University of Nicosia Medical School, Nicosia, Cyprus; 11 School of Medicine, Lebanese American University, Byblos, Lebanon; 12 Stroke Unit, Service de Neurologie, CHU Henri Mondor- 94010, Créteil Cedex, France; 13 UPE-C, Faculté de Santé, Université Paris-Est Créteil, Paris, France; 14 INSERM U955-E01, IMRB, Créteil, France

**Keywords:** stroke; knowledge; awareness

## Abstract

**Background:** Evaluation of the knowledge about stroke in the general population is extremely vital as it prevents stroke development, limits complications, and achieves better quality of life.  We assume that the general Lebanese population lacks awareness about stroke and its associated complications. This study aims to evaluate stroke knowledge and determine the factors associated with stroke awareness among the general Lebanese population.

**Methods:** This cross-sectional study assessed respondents’ sociodemographic characteristics and the identification of risk factors, warning signs, stroke consequences, and early response to stroke symptoms. A total of 551 Lebanese adults without a history of stroke filled in an online self-reported questionnaire publicly shared on social applications. Logistic regression analysis was performed to identify the factors associated with poor knowledge of stroke.

**Results:** Among the 551 participants enrolled, 403 (74.2%) were females and 312 (56.7%) were under 30 years of age. Females compared to males and employed compared to unemployed had significantly higher odds of identifying at least one risk factor (OR=4.3 [95%CI=1.1;16.8] and 6 [95%CI=1.2;29.6], respectively). Also, when compared to unemployed, employed participants had significantly higher odds of recognizing at least one of the early stroke symptoms (OR=3.3 [95%CI=1.2;8.9]) and identifying at least one of the stroke consequences (OR=5.3 [95%CI=1.1;25.9]). Reaching a university level of education compared to a school level was associated with significantly higher odds (OR=2.3 [95%CI=1.1;4.8]) of taking a patient to a hospital.

**Conclusion:** Well-educated, employed, and female participants were more knowledgeable about stroke. Tailored interventions focusing on individuals with inadequate stroke literacy are needed. Further studies, more representative of the general Lebanese population with a larger sample size, are necessary to confirm our findings.

## Introduction

Ischemic stroke is neurologic dysfunction caused by sudden embolic occlusions in the cerebral vessel. ^1^^,^^2^ It accounts for around 87% of all cases of stroke worldwide and is a major contributing cause of mortality and a significant factor of disability in adults. ^3^^,^^4^ In Lebanon, the adjusted prevalence of stroke is 0.5%, and the cumulative mortality rates are 14.1% at one month. ^5^^,^^6^

Primary prevention against stroke is considered a cornerstone in minimizing stroke development. It is reached through a variety of strategies that focus on identifying related risk factors, implementing preventative measures, and educating patients. Awareness programs should aim to increase community knowledge, which is one of the most effective prevention measures; this raises the need for an accurate assessment of stroke knowledge and its related triggers. ^7^^–^^11^ Moreover, knowledge will not only improve patients’ quality of life but can also prevent health care professionals from being overwhelmed when stroke cases arrive in the hospital at an early stage. ^12^ This is noteworthy as 80% of stroke cases are preventable if adequate precautions and measures are taken promptly. ^13^

Therefore, it is essential to explore features such as lifestyle, behavior, ^14^ educational background, smoking history, ^15^ and socioeconomic status ^15^^,^^16^ to understand the disparities in stroke knowledge between different sociodemographic groups. Since stroke risk factors (e.g., history of hypertension, diabetes) are identifiable in individuals with low socioeconomic, past medical history is also an important factor to investigate. Educational level, personal history of smoking, and high-income status have been associated with increased stroke knowledge. ^17^ With regard to gender, there are conflicting results. Some studies reported that females are more likely than males to present non-traditional stroke warning signs, develop stroke, and arrive late to the hospital ^18^^–^^20^; on the contrary, other studies showed that females are able to identify all the five conventional warning signs of a stroke and quickly call the emergency line. ^21^

In Lebanon, various studies were conducted that assessed stroke risk factors, prevalence, adherence to post-discharge medications, and acute hypertension treatment. ^5^^,^^6^ However, no nationwide study has been conducted yet. Only one study assessed the public awareness towards stroke but examined a small population, as it was limited to the capital city. ^22^ Thus, this study aims to evaluate stroke knowledge and determine the factors associated with stroke awareness among the general Lebanese population.

## Methods

### Ethical approval

The study was conducted based on the declaration of Helsinki and was approved by the ethics committee at the School of Pharmacy of the Lebanese International University (202ORC-035-LIUSOP). Written informed consent was obtained from all participants before inclusion in the study.

### Study design and procedure

This descriptive observational cross-sectional study was carried out from September 2020 through January 2021 on the Lebanese population from all Governorates (Beirut, Mount Lebanon, North, South, and Beqaa), using an anonymous online survey. A snowball sampling method was used to abide by the lockdown restrictions enforced by the Lebanese Government. An electronic survey was developed using Google forms platform and was distributed through different social media platforms (i.e., WhatsApp, LinkedIn, and Facebook). The link to the questionnaire was posted by the authors on each platform and made available to all the users, who are given the right to share. No particular group was targeted. Participation in this survey was voluntary and free of charge. Participants over the age of 18 were eligible, while those with a history of stroke were excluded. The anonymity of the participants was guaranteed during the data collection process.

### Sample size calculation

Based on another study, which concluded that around 71.8% of the participants were able to identify at least three out of five stroke risk factors, ^23^ and in the absence of similar studies in Lebanon, the Epi Info software version 7.2 (population survey; https://www.cdc.gov/epiinfo/index.html) calculated a minimum sample of 312 participants at a confidence level of 95%. The purpose for oversampling is to take into account patients’ refusal.

### Questionnaire

The questionnaire was distributed in Arabic, the native language of Lebanon. ^24^ The questionnaire was structured initially in the English language and then translated by a single bilingual translator, whose native language is Arabic and fluent in English. An expert committee formed by healthcare professionals and a language professional verified the Arabic-translated version. A backward translation was then performed by a native English speaker translator, fluent in Arabic and unfamiliar with the concepts of stroke. The back-translated English questionnaire was subsequently compared with the original English one, by the expert committee, aiming to discern discrepancies and to solve any inconsistencies between the two versions. The process of forward-back translation was repeated until all ambiguities disappeared. It was self-administered and required approximately 20 minutes to be completed.

The questionnaire is similar to those used in the literature. ^22^^,^^23^^,^^25^^–^^32^ The current questionnaire, methods, and tools used in this study was mainly adapted from a study conducted in Jordan in which the general knowledge about stroke was assessed. ^33^ The questionnaire was structured similarly to the one of Jordan in all the aspects that covered stroke knowledge except for the sociodemographic factors (i.e., economic status and residence area) because of the discrepancy between the two countries. However, differences were only in the sociodemographic characteristics due to the slight variation between the two countries. The questionnaire was structured to collect information about stroke in terms of symptoms, risk factors, early warning signs, and complications. Participants completed it without the assistance of investigators to avoid any potential influence when responding to questions. The opening section of the questionnaire covered the sociodemographic characteristics, including age, marital status, smoking status (positive when participant smoked for at least a year), employment status (employed versus unemployed), monthly income, residence (urban versus rural), educational level, and past medical history determined by self-report such as ever being diagnosed with the medical condition by a healthcare professional (e.g., hypertension, diabetes mellitus, dyslipidemia). Age was classified into four categories (18-29, 30-49, 50-70, and above 70 years) while family income was divided into three categories: low (<1,500,000 Lebanese Lira (LL)), intermediate (1,500,000-3,000,000 LL), and high (>3,000,000 LL). ^24^^,^^29^

The second section evaluated the general knowledge related to stroke. Participants responded to whether stroke is a disease that: 1) affects the brain, 2) is an old person disease, 3) is contagious, 4) is hereditary, and 5) and can be prevented. Also, this section assessed awareness of the risk factors of stroke, including old age, hypertension, diabetes mellitus, heart disease, high cholesterol, smoking, alcohol consumption, physical inactivity, obesity, and stress. Furthermore, this section focused on participants’ knowledge of early stroke warning signs including: 1) sudden numbness or weakness of the face, arms, or legs, 2) sudden difficulty speaking or understanding speech, 3) sudden blurry vision or visual impairment in one or both eyes, 4) sudden dizziness, or loss of balance or coordination, and 5) sudden severe headache. Additionally, participants reported potential consequences of stroke: 1) movement and functional problems (i.e., one-sided paralysis, loss of ability to walk, tiredness, fatigue), 2) Cognitive and memory problems (i.e., loss of ability to speak, write, read, remember or understand), 3) visual problems (i.e., loss of sight or blurred vision), 4) emotional and personality changes (i.e., depression, anger, mood changes), and 5) long-term disabilities. Three questions assessed the attitude and the reaction of people towards a patient experiencing stroke (e.g., willingness to take a patient to hospital care); two others were on the curiosity and self-assessment while the last one was to determine the sources of information of knowledge about stroke. Participants were given one point for each correct response to the above statements (see extended data – date key). ^24^ Missing answers were not counted. ^25^ Sometimes, multiple answers were allowed; that is why the total score was higher than the total number of questions.

### Statistical analysis

Data collected were analyzed using the Statistical Package for Social Sciences version 25.0 (SPSS; https://www.ibm.com/be-en/products/spss-statistics). A freely accessible software alternative software to run this analysis is RStudio ( https://www.rstudio.com/products/rstudio/download/). Continuous variables were presented as mean standard deviation and 95% confidence interval. Categorical and ordinal variables were shown as frequencies (n) and percentages (%). Correlations between risk factors, early symptoms, and consequences of stroke with the socio-demographics and past medical history were determined by the Pearson chi-square or Fisher’s exact test if the cell count was less than five.

Logistic regressions models were used to assess the association between sociodemographic factors, the medical history that showed a P<0.2 in the bivariate analysis and identified a total number of risk factors, identified a total number of early symptoms, identified a total number of consequences, and willingness to take a patient to a hospital. Potential confounders were eliminated if P>0.2 to protect against residual confounding.

The results were presented in the form of odds ratios (OR) and 95% confidence interval. Statistical tests were two-tailed and indicated statistical significance at P<0.05.

## Results

### Sample description

Out of the total 551 participants enrolled in the study, 403 (74.2%) were females, 312 (56.7%) were under 30 years of age, and almost half were single and residing in rural areas. The most common concomitant disease was dyslipidemia (17.6%), followed by obesity (16.8%) and peptic ulcer (16.2%). The sociodemographic factors results are displayed in Table 1. ^24^ Almost all the participants had heard of stroke (93.6%); 69.1% know about stroke if someone around had the disease.

**Table 1.** Participants’ socio-demographic characteristics, past medical history, and familiarity with stroke.

Variables (N=551)	Categories	Frequency (%)
**Socio-demographic characteristics**		
Gender	Male	140 (25.8)
	Female	403 (74.2)
Age (years)	<30	312 (56.7)
	30-49	175 (31.8)
	>50	63 (11.5)
Residence area	Urban	256 (46.8)
	Rural	291 (53.2)
Marital status	Single	274 (50.1)
	Married	254 (46.4)
	Divorced	12 (2.2)
	Widowed	7 (1.3)
Education (years)	School (maximum 12)	53 (9.6)
	University (minimum 13)	497 (90.4)
Employment status	Unemployed	246 (44.9)
	Employed	302 (55.1)
Income level	Low (<1,500,000 LL *)	199 (43.2)
	Medium (1,500,000-3,000,000 LL *)	206 (44.7)
	High (>3,000,000 LL *)	56 (12.1)
History of smoking (≥1 year)	Yes	147 (27.4)
Past medical history	Hypertension	64 (12.3)
	Diabetes mellitus	37 (7.2)
	Dyslipidemia	91 (17.6)
	Arrhythmia	81 (15.7)
	Kidney disease	22 (4.3)
	Peptic ulcer	82 (16.2)
	Depression	73 (14.2)
	Obesity	87 (16.8)
Familiarity with stroke	Ever heard of stroke	511 (93.6)
	History of stroke in the family	150 (27.8)
	Personally know someone with stroke	378 (69.1)

* LL, Lebanese Lira.

### Stroke knowledge, risk factors, early warning signs, consequences, and sources of information

**Figure 1.**
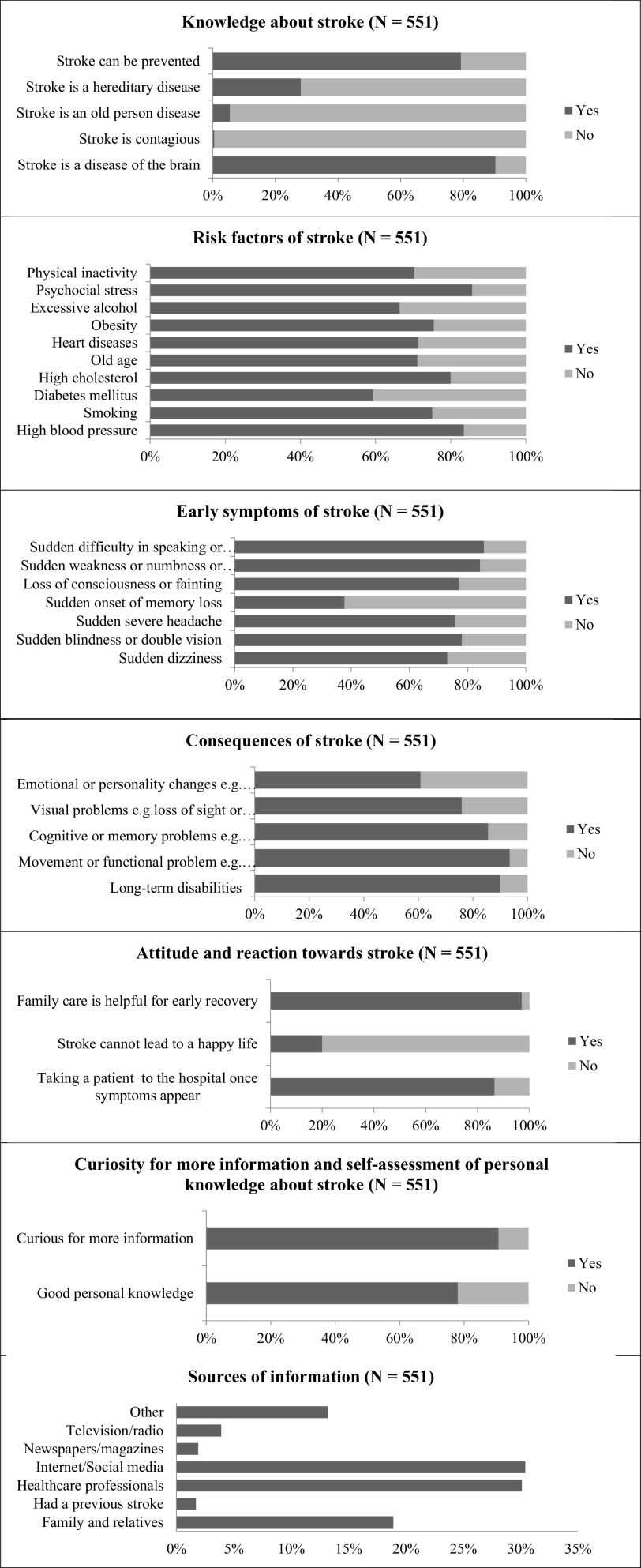
Percentages (%) of responses regarding stroke’ knowledge, risk factors, early symptoms, attitude and reaction toward stroke, consequences, and sources of information.

Our sample revealed a variable level of knowledge about stroke ( Figure 1 and Table 2). The majority were aware that stroke is a brain disease and that it can be prevented (80% and 90%, respectively). Approximately half of the participants could identify four out of five correct answers related to stroke knowledge. Furthermore, 90% believed that psychosocial stress was the most common risk factor of stroke, followed by hypertension and dyslipidemia. The most common warning signs were ‘Sudden difficulty in speaking or understanding speech’ and ‘Sudden weakness/numbness of the arms/face/legs’, accounting for 85%. Only 26% identified all the risk factors, 22.7% recognized all the symptoms, and 44% stated all possible stroke consequences. Internet/social media (30%), health-care professionals (30%), and family/relatives (18.9%) were the main sources of information of knowledge about stroke. It is noteworthy that missing answers were not counted in the analysis.

**Table 2.** Number of stroke risk factors, early symptoms, and consequences that were identified by the participants.

Variables (n=551)	Categories	Frequency (%)	Cumulative frequency (%) *
Number of correct answers regarding stroke in the general knowledge	Less than two	0 (0)	0 (0)
	Two	7 (1.3)	7 (1.3)
	Three	52 (9.6)	59 (10.9)
	Four	225 (41.6)	284 (52.5)
	Five	257 (47.5)	541 (100)
Number of identified risk factors of stroke	Zero	12 (2.2)	12 (2.2)
	One	9 (1.7)	21 (3.9)
	Two	11 (2)	32 (5.9)
	Three	20 (3.7)	52 (9.5)
	Four	34 (6.2)	86 (15.8)
	Five	30 (5.5)	116 (21.3)
	Six	63 (11.6)	179 (32.8)
	Seven	68 (12.5)	247 (45.3)
	Eight	78 (14.3)	325 (59.6)
	Nine	78 (14.3)	403 (73.9)
	Ten	142 (26.1)	545 (100)
Number of identified early symptoms of stroke	Zero	22 (4.1)	22 (4.1)
	One	14 (2.6)	36 (6.6)
	Two	13 (2.4)	49 (9)
	Three	57 (10.5)	106 (19.6)
	Four	74 (13.7)	180 (33.2)
	Five	117 (21.6)	297 (54.8)
	Six	122 (22.5)	419 (77.3)
	Seven	123 (22.7)	542 (100)
Number of identified consequences of stroke	Zero	12 (2.2)	12 (2.2)
	One	16 (2.9)	28 (5.2)
	Two	35 (6.4)	63 (11.6)
	Three	74 (13.6)	137 (25.2)
	Four	167 (30.8)	304 (56)
	Five	239 (44)	543 (100)

* Missing answers were not included in the analysis.

### Bivariate analysis

A significantly higher percentage of females versus males (74.9% vs. 25.1%), residents of the rural versus urban areas (53.8 % vs. 46.2%), and employed versus unemployed (99% vs. 96.3%) correctly identified risk factors. Moreover, a significantly higher percentage of participants who had a job versus unemployed (97.7% vs. 93.7%) recognized at least one warning symptom of stroke. A significantly higher percentage of subjects with university level of education compared to school level (91.5 % vs. 8.5%) and those employed versus unemployed (99% vs. 96.2%) correctly identified the consequences emerging from stroke ( Table 3).

**Table 3.** Association of risk factors, early symptoms, and consequences of stroke with the socio-demographic characteristics and past medical history.

Variables (N=551)		Risk factor(s) identified (≥1)			Early symptom(s) identified (≥1)			Consequence(s) identified (≥1)		
		Yes (n=533), n (%)	No (n=12), n (%)	P-value	Yes (n= 520), n (%)	No (n=22), n (%)	P-value	Yes (n= 520), n (%)	No (n=22), n (%)	P-value
**Socio-demographic characteristics**										
Gender	Male	132 (25.1)	7 (58.3)	0.016 ^†^	129 (25)	8 (40)	0.133	134 (25.6)	6 (50)	0.089
	Female	394 (74.9)	5 (41.7)		386 (75)	12 (60)		390 (74.4)	6 (50)	
Age (years)	<30	302 (56.7)	6 (50)	0.832	294 (56.5)	12 (54.5)	0.880	300 (56.5)	6 (50)	0.698
	30-49	169 (31.7)	5 (41.7)		166 (31.9)	8 (36.4)		170 (32)	4 (33.3) ^°^	
	> 50	62 (11.6)	1 (8.3) ^°^		60 (11.5)	2 (9.1) ^°^		61 (11.5)	2 (16.7) ^°^	
Residence area	Urban	245 (46.2)	9 (75)	0.048 ^†^	240 (46.4)	14 (63.6)	0.113	246 (46.6)	6 (50)	0.815
	Rural	285 (53.8)	3 (25) ^°^		277 (53.6)	8 (36.4)		282 (53.4)	6 (50)	
Marital status	Single	263 (49.6)	8 (66.7)	0.602	257 (49.7)	12 (54.5)	0.368	261 (49.4)	8 (66.7)	0.602
	Married	248 (46.8)	4 (33.3) ^°^		242 (46.8)	9 (40.9)		248 (47)	4 (33.3) ^°^	
	Divorced	12 (2.3)	0 (0) ^°^		12 (2.3)	0 (0) ^°^		12 (2.3)	0 (0) ^°^	
	Widowed	7 (1.3)	0 (0) ^°^		6 (1.2)	1 (4.5) ^°^		7 (1.3)	0 (0) ^°^	
Educational level	School	48 (9)	3 (25) ^°^	0.093	47 (9)	3 (13.6) ^°^	0.445	45 (8.5)	5 (41.7)	0.003 ^†^
	University	485 (91)	9 (75)		473 (91)	19 (86.4)		486 (91.5)	7 (58.3)	
Employment status	Unemployed	233 (96.3)	9 (3.7)	0.032 ^†^	223 (93.7)	15 (6.3)	0.020 ^†^	230 (96.2)	9 (3.8)	0.030 ^†^
	Employed	298 (99)	3 (1) ^°^		295 (97.7)	7 (2.3)		299 (99)	3 (1) ^°^	
Income level	Low (<1,500,000 LL *)	196 (43.4)	2 (28.6) ^°^	0.340	187 (42.6)	7 (41.2)	0.660	194 (43.2)	3 (33.3) ^°^	0.892
	Medium (1,500,000-3,000,000 LL *)	202 (44.7)	3 (42.9) ^°^		197 (44.9)	9 (52.9)		201 (44.8)	5 (55.6)	
	High (>3,000,000 LL *)	54 (11.9)	2 (28.6) ^°^		55 (12.5)	1 (5.9) ^°^		54 (12)	1 (11.1) ^°^	
History of smoking (≥1 year)	No	383 (73)	5 (50)	0.147	374 (73)	14 (70)	0.764	383 (73)	6 (54.5)	0.183
	Yes	142 (27)	5 (50)		138 (27)	6 (30)		142 (27)	5 (45.5)	
**Past medical history**										
Hypertension	No	448 (87.8)	8 (80)	0.354	435 (87.3)	19 (95)	0.493	447 (87.6)	10 (90.9)	1.000
	Yes	62 (12.2)	2 (20) ^°^		63 (12.7)	1 (5) ^°^		63 (12.4)	1 (9.1) ^°^	
Diabetes Mellitus	No	465 (92.8)	9 (90)	0.532	454 (92.7)	18 (94.7)	1.000	464 (92.6)	11 (100)	1.000
	Yes	36 (7.2)	1 (10) ^°^		36 (7.3)	1 (5.3) ^°^		37 (7.4)	0 (0) ^°^	
Dyslipidemia	No	417 (82.2)	9 (90)	1.000	406 (82)	18 (90)	0.551	416 (82.1)	11 (100)	0.226
	Yes	90 (17.8)	1 (10) ^°^		89 (18)	2 (10) ^°^		91 (17.9)	0 (0) ^°^	
Arrhythmia	No	424 (84.1)	9 (90)	1.000	414 (84.1)	17 (85)	1.000	424 (84.1)	10 (90.9)	1.000
	Yes	80 (15.9)	1 (10) ^°^		78 (15.9)	3 (15) ^°^		80 (15.9)	1 (9.1) ^°^	
Kidney disease	No	475 (95.6)	10 (100)	1.000	463 (95.5)	20 (100)	1.000	475 (95.6)	11 (100)	1.000
	Yes	22 (4.4)	0 (0) ^°^		22 (4.5)	0 (0) ^°^		22 (4.4)	0 (0) ^°^	
Peptic ulcer	No	416 (83.9)	8 (80)	0.669	407 (84.1)	15 (75)	0.347	417 (83.9)	8 (80)	0.668
	Yes	80 (16.1)	2 (20) ^°^		77 (15.9)	5 (25)		80 (16.1)	2 (20) ^°^	
Depression	No	429 (85.5)	10 (100)	0.371	422 (86.3)	16 (80)	0.504	430 (85.7)	10 (90.9)	1.000
	Yes	73 (14.5)	0 (0) ^°^		67 (13.7)	4 (20) ^°^		72 (14.3)	1 (9.1) ^°^	
Obesity	No	420 (83)	9 (90)	1.000	409 (82.8)	18 (90)	0.551	419 (82.8)	11 (100)	0.225
	Yes	86 (17)	1 (10) ^°^		85 (17.2)	2 (10) ^°^		87 (17.2)	0 (0) ^°^	

^°^ Fisher’s exact test was used when the cell counts were less than 5.

^†^ Significant P-values.

* LL, Lebanese Lira

A significantly higher number of correct answers was associated with university compared to school level of education (91.8% vs. 8.2%), no history of dyslipidemia compared to having dyslipidemia (83.8% vs. 16.2%), and no history of depression versus having depression (87.2% vs. 12.8%) ( Table 4).

**Table 4.** Association of taking a patient who is experiencing stroke to the hospital with socio-demographic characteristics, and past medical history.

Variables (N=551)		Taking a patient who is experiencing stroke to the hospital)		
		Yes (n=473), n (%)	No (n=74), n (%)	P-value
**Socio-demographic characteristics**				
Gender	Male	117 (25)	23 (31.9)	0.211
	Female	351 (75)	49 (68.1)	
Age (years)	<30	263 (55.6)	46 (62.2)	0.475
	30-49	153 (32.3)	22 (29.7)	
	>50	57 (12.1)	6 (8.1)	
Residence area	Urban	214 (45.3)	41 (56.9)	0.066 ^†^
	Rural	258 (54.7)	31 (43.1)	
Marital status	Single	237 (50.3)	35 (47.9)	0.567
	Married	216 (45.9)	37 (50.7)	
	Divorced	12 (2.5)	0 (0) ^°^	
	Widowed	6 (1.3)	1 (1.4) ^°^	
Educational level	School	39 (8.2)	13 (17.6)	0.011 ^†^
	University	434 (91.8)	61 (82.4)	
Employment status	Unemployed	207 (85.2)	36 (14.8)	0.383
	Employed	265 (87.7)	37 (12.3)	
Income level	Low (<1,500,000 LL *)	174 (43)	25 (44.6)	0.932
	Medium (1,500,000-3,000,000 LL *)	181 (44.7)	25 (44.6)	
	High (>3,000,000 LL *)	50 (12.3)	6 (10.7)	
History of smoking (≥1 year)	No	336 (72.4)	53 (73.6)	0.832
	Yes	128 (27.6)	19 (26.4)	
**Past medical history**				
Hypertension	No	400 (87.9)	57 (86.4)	0.720
	Yes	55 (12.1)	9 (13.6)	
Diabetes Mellitus	No	414 (92.8)	61 (92.4)	0.803
	Yes	32 (7.2)	5 (7.6)	
Dyslipidemia	No	377 (83.8)	50 (73.5)	0.038 ^†^
	Yes	73 (16.2)	18 (26.5)	
Arrhythmia	No	379 (84.6)	55 (82.1)	0.599
	Yes	69 (15.4)	12 (17.9)	
Kidney disease	No	423 (95.7)	63 (95.5)	1.000
	Yes	19 (4.3)	3 (4.5) ^°^	
Peptic ulcer	No	373 (84)	52 (82.5)	0.767
	Yes	71 (16)	11 (17.5)	
Depression	No	389 (87.2)	51 (76.1)	0.015 ^†^
	Yes	57 (12.8)	16 (23.9)	
Obesity	No	374 (82.9)	56 (84.8)	0.697
	Yes	77 (17.1)	10 (15.2)	

^°^ Fisher’s exact test was used when the cell counts were less than 5.

^†^ Significant p-values.

* LL, Lebanese Lira

### Multivariable analysis

When considering the identification of at least one risk factor as the dependent variable, our analysis showed that females compared to males and employed compared to unemployed had significantly higher odds (OR 4.3 [95% CI 1.1;16.8] and 6 [95% CI 1.2;29.6], respectively).

While when considering the identification of at least one early stroke symptom as the dependent variable, employed compared to unemployed had significantly higher odds (OR 3.3 [95% CI 1.2;8.9]).

Also, when considering the identification of at least one stroke consequence as the dependent variable, employed compared to unemployed had significantly higher odds (OR 5.3 [95% CI 1.1;25.9]).

When taking the identification of at least one stroke symptom as the dependent variable, university level compared to school level of education had significantly higher odds (OR 2.3 [95% CI 1.1;4.8]) ( Table 5).

**Table 5.** Multivariate analysis.

Variables (N=551)	β (SE)	OR (95% CI)	P-value
**Risk factor(s) identified (≥1)**			
Gender (female versus male *)	1.5 (0.7)	4.3 (1.1; 16.8)	0.034 ^†^
Residence area (rural versus urban *)	1 (0.7)	2.8 (0.7; 11.1)	0.149
Educational level (university versus school *)	0.3 (0.8)	1.3 (0.3; 6.9)	0.729
Employment status (employed versus unemployed *)	1.8 (0.8)	6 (1.2; 29.6)	0.028 ^†^
History of smoking (≥1 year) (yes versus no *)	-0.6 (0.7)	0.5 (0.1; 2.1)	0.366
**Early symptom(s) identified (≥1)**			
Gender (female versus male *)	0.8 (0.5)	2.3 (0.9; 5.8)	0.086
Residence area (rural versus urban *)	0.6 (0.5)	1.7 (0.7; 4.4)	0.241
Employment status (employed versus unemployed *)	1.2 (0.5)	3.3 (1.2; 8.9)	0.016 ^†^
**Consequence(s) identified (≥1)**			
Gender (female versus male *)	0.8 (0.7)	2.3 (0.6; 8.4)	0.214
Educational level (university versus school *)	1.3 (0.7)	3.5 (0.9; 13.1)	0.06
Employment status (employed versus unemployed *)	1.7 (0.8)	5.3 (1.1; 25.9)	0.04 ^†^
History of smoking (≥1 year) (yes versus no *)	-0.6 (0.7)	0.6 (0.2; 2.1)	0.404
**Taking a patient to a hospital**			
Residence area (rural versus urban *)	0.4 (0.3)	1.5 (0.9; 2.6)	0.136
Educational level (university versus school *)	0.8 (0.4)	2.3 (1.1; 4.8)	0.032 ^†^
Dyslipidemia (yes versus no *)	-0.4 (0.3)	0.7 (0.3; 1.3)	0.244
Depression (yes versus no *)	-0.6 (0.3)	0.5 (0.3; 1)	0.067

Abbreviations: β, Beta; SE, standard error; OR; odds ratio; CI, confidence interval.

Dependent variables: identification of stroke risk factors, stroke early symptoms, stroke consequences, taking a patient who is experiencing stroke to the hospital.

Independent variables: sociodemographic factors (gender, residence area, educational level, employment status, and smoking status).

^†^ Significant P-values.

^*^ Reference.

## Discussion

The current study evaluated factors related to knowledge about stroke risk factors, early symptoms, and consequences in a sample of the general Lebanese population without a history of stroke. The results indicated that the majority of participants identified at least one stroke related risk factor, symptom, and consequence. Our percentages are higher than those reported in the literature, showing that at least one stroke risk factor may be identified by more than half of the sample, probably as most of our participants had a university degree. ^7^^,^^34^^–^^36^ A Lebanese study among 390 participants showed that 68% could spontaneously recall at least one stroke symptom, and 85.4% spontaneously recalled at least one risk factor. ^22^ A study done in Spain has found that 60% of the sample identified at least one risk factor, ^37^ and an Australian study revealed that 76% could name at least one risk factor. ^7^ However, other studies have demonstrated a low knowledge of stroke risk factors and symptoms in the general population. ^38^^–^^40^

Our results revealed that hypertension and psychological stress were the most known risk factors, with a percentage close to 80%. A previous study in Lebanon had found that the most recalled risk factor was hypertension (48.2%), followed by stress (43.1%). ^22^ A study in Morocco among 469 participants has found similar results, with a percentage close to 50%. These findings are in line with those of several surveys conducted in different countries. Although diabetes is a major risk factor for stroke, ^41^ it was the least reported in our study. Similarly, another study found that more than half of the sample did not recognize diabetes or hypercholesterolemia as risk factors for stroke. ^42^

Also, our results showed a higher percentage of participants recalling at least one stroke early symptom compared to those studies in Portugal (74.2%), ^43^ Norway (70.7%), ^44^ Oman (68%), ^45^ Korea (65%), ^46^ and Jordan (95.5%). ^33^ Sudden loss of speech was the most frequently reported stroke symptom in our study compared to studies in Jordan (54.7%), Ireland (54%), ^47^ and Australia (60.1%). ^7^ However, other studies reported sudden weakening of one side of the body to be the most prevalent symptom, as in the Omani (65 %) ^45^ and Nigerian (55 %) populations. ^48^

In our study, most of the sample was aware of the importance of going to a hospital emergency as early as possible after a stroke is identified, in agreement with previous findings showing that a high percentage of participants recognized the need for immediate medical care. ^22^ A study in Oman among 400 participants found that 73% would go immediately to the hospital if they knew they had a stroke. ^45^ However, the percentage found in international studies was lower, with only 47% claiming they would go to a hospital if they suspected they had a stroke. ^38^

The adequate knowledge of stroke risk factors, symptoms, and consequences in our sample could be explained by the young age of the participants and the high level of education, which might be related to better awareness of these aspects of the stroke.

Our results showed that females had better knowledge about stroke risk factors than males, in agreement with other findings. ^36^^,^^49^^,^^50^ However, some studies did not detect any gender differences ^51^^,^^52^ in risk factors awareness, and others showed a better knowledge among men. ^37^^,^^48^ Women tend to be more knowledgeable and might be more interested in health topics than men and take more time to seek related information. ^53^ In this regard, the country of origin is an essential factor to consider because of cultural gaps in gender distribution, access to education, and information in each country. ^39^

Moreover, our results revealed that being employed was significantly associated with better awareness of stroke risk factors, early symptoms, and consequences. Similarly, a study in Spain among 2,411 persons has found that actively employed individuals have a better knowledge of stroke than unemployed. ^54^ One possible explanation could be that employed people might have the financial capacity to access information or visit their physician more regularly for check-ups. Our results also showed that higher education levels were a significant factor associated with the need for immediate medical intervention and direct transfer to the hospital after warning signs of stroke. Expectedly, the more literate the participants, the more health-related knowledge they have, making them more ready to respond to any stroke condition.

### Limitations

This study has several limitations. The results could not be representative of the entire Lebanese population as the majority of participants were females, well-educated with computer literacy and internet access; thus, less-educated people and those who did not have access to a computer or mobile or internet were not assessed. Additionally, its cross-sectional design cannot infer causality. Information bias could also exist as the study questionnaire was online and answers were self-reported. The answers to stroke awareness might be overestimated because the questionnaire used consisted of multiple-choice questions with limited options available; thus, the participants could have guessed the answers. Selection bias might have also occurred since the sample was not randomly selected but rather gathered using the snowball sampling technique. Residual confounding bias is also possible since there might be factors related to stroke awareness that were not measured in this study.

## Conclusion

The evaluation of stroke knowledge among the general Lebanese population showed that well-educated, employed, and female participants were more knowledgeable about stroke. Tailored interventions focusing on individuals with inadequate stroke literacy are needed to improve stroke awareness.

Further studies, more representative of the general Lebanese population and with a larger sample size, are necessary to confirm our findings.

## Data availability

### Underlying data

OSF: Factors Associated with Knowledge and Awareness of Stroke Among the Lebanese Population: A Cross-Sectional Study. https://doi.org/10.17605/OSF.IO/Y2DAP. ^24^

This project contains the following underlying data:

- Anonymous data_Stroke Education Article_Dr Diana Malaeb.sav

Data are available under the terms of the Creative Commons Attribution 4.0 International license (CC-BY 4.0).

### Extended data

OSF: Factors Associated with Knowledge and Awareness of Stroke Among the Lebanese Population: A Cross-Sectional Study. https://doi.org/10.17605/OSF.IO/Y2DAP. ^24^

This project contains the following extended data:

- Questionnaire_Stroke Education Article_Dr Diana Malaeb.doc. (English version of the questionnaire).
- Questionnaire_Stroke Education Article_Dr Diana Malaeb.Arabic.doc. (Arabic version of the questionnaire).
- Data key_Stroke Education Article_Dr Diana Malaeb.docx.

Data are available under the terms of the Creative Commons Attribution 4.0 International license (CC-BY 4.0).
