# Supplementary material for: Factors associated with knowledge and awareness of stroke among the Lebanese population: A cross-sectional study
Source: F1000Res. 2022 Jul 11;11:425. Originally published 2022 Apr 14. [Version 2] doi: 10.12688/f1000research.108734.2 (PMC9160706; doi:10.12688/f1000research.108734.2)
Supplement: Peer review history

# Peer review: Reviewer response for version 1

**DOI:** 10.5256/f1000research.120152.r135285

**Published:** 2022-06-01

**Authors:** Ammar ali saleh Jaber

**Affiliations:** 1 Department of Clinical Pharmacy & Pharmacotherapeutics, Dubai Pharmacy College for Girls, Dubai, United Arab Emirates

This research assesses the knowledge and awareness of the Lebanese population regarding stroke in a cross-sectional study. The methodology is scientifically founded. The article is well-written and raises an interesting question. The results are represented adequately. The study paves the road for awareness campaigns about stroke. This article is of good quality, fit for indexing and citation. 

 However, I have some minor points for revision before final indexing:

1. State the objectives of the study in a clearer manner.
2. The real questionnaire should be attached as supplement material  for reproducibility.
3. Some of the English writing in the intro needs to be revised before submission.
4. In the 4th paragraph of the intro, a very brief comparison of Lebanon people to a neighbouring country might be needed.

Overall, this manuscript will be very interesting after doing the above recommendations and insertion of the recommended citations.

Is the work clearly and accurately presented and does it cite the current literature?

Yes

If applicable, is the statistical analysis and its interpretation appropriate?

Yes

Are all the source data underlying the results available to ensure full reproducibility?

Yes

Is the study design appropriate and is the work technically sound?

Yes

Are sufficient details of methods and analysis provided to allow replication by others?

Yes

Reviewer Expertise:

NA

I confirm that I have read this submission and believe that I have an appropriate level of expertise to confirm that it is of an acceptable scientific standard.

# Response

**Published:** 2022-06-10

**Authors:** Nada Dia

**Affiliations:** lebanese international university, Lebanon

Dear Prof. Ammar ali saleh Jaber,

 Thank you for reviewing our article.

 Our reply is stated below each question:

1. State the objectives of the study in a clearer manner.
  Thank you for pointing this out. We have stated the objectives clearly, as requested, in the second version.
2. The real questionnaire should be attached as supplement material for reproducibility.
  Thank you for this suggestion. Abiding by the policy of the journal, the questionnaire is accessible online via the link provided in the section on data availability.
3. Some of the English writing in the intro needs to be revised before submission.
  Thank you for pointing this out. English writing was revised and corrected.
4. In the 4th paragraph of the intro, a very brief comparison of Lebanon people to a neighboring country might be needed.
  Thank you for raising an important point here. The introduction was expanded to cover neighboring countries.

We look forward to hearing from you in due time regarding our second version and to respond to any further questions and comments you may have.

 Sincerely,

 Nada Dia

# Peer review: Reviewer response for version 1

**DOI:** 10.5256/f1000research.120152.r135286

**Published:** 2022-04-22

**Authors:** Mohammad Al-Shorbagy

**Affiliations:** 1 Department of Pharmacology and Toxicology, Faculty of Pharmacy, Cairo University, Cairo, Egypt

The current cross-sectional study highlights the factors associated with the knowledge and awareness of stroke among a sample of the Lebanese population. Motives to initiate the study in that population seem logical and the stratification of the population groups serves the purpose of the study. Results are coherent and neatly described with appropriate statistical analysis. The conclusions drawn are not overly expressed and are matching with the obtained results and can drive action to population awareness as well as feed future research in that particular setup.

Is the work clearly and accurately presented and does it cite the current literature?

Yes

If applicable, is the statistical analysis and its interpretation appropriate?

Yes

Are all the source data underlying the results available to ensure full reproducibility?

Yes

Is the study design appropriate and is the work technically sound?

Yes

Are the conclusions drawn adequately supported by the results?

Yes

Are sufficient details of methods and analysis provided to allow replication by others?

Yes

Reviewer Expertise:

Neuropharmacology

I confirm that I have read this submission and believe that I have an appropriate level of expertise to confirm that it is of an acceptable scientific standard.

# Response

**Published:** 2022-06-10

**Authors:** Nada Dia

**Affiliations:** lebanese international university, Lebanon

Dear Prof. Mohammad Al-Shorbagy

 Thank you for reviewing our article.

 We look forward to hearing from you in due time regarding our second version and to responding to any further questions and comments you may have.

 Sincerely,

 Nada Dia
